# Endoscopic Bridge-and-Seal of Bile Leaks Using a Fully Covered Self-Expandable Metallic Stent above the Papilla

**DOI:** 10.3390/jcm11206019

**Published:** 2022-10-12

**Authors:** Koshiro Fukuda, Yousuke Nakai, Suguru Mizuno, Tatsuya Sato, Kensaku Noguchi, Sachiko Kanai, Tatsunori Suzuki, Ryunosuke Hakuta, Kazunaga Ishigaki, Kei Saito, Tomotaka Saito, Naminatsu Takahara, Tsuyoshi Hamada, Hirofumi Kogure, Mitsuhiro Fujishiro

**Affiliations:** 1 Department of Gastroenterology, Graduate School of Medicine, The University of Tokyo, Tokyo 113-8655, Japan; 2 Department of Endoscopy and Endoscopic Surgery, The University of Tokyo Hospital, Tokyo 113-8655, Japan; 3 Department of Gastroenterology and Hepatology, Saitama Medical University Hospital, Saitama 350-0495, Japan; 4 Department of Gastroenterology and Hepatology, Nihon University Itabashi Hospital, Tokyo 173-8610, Japan

**Keywords:** postoperative bile leaks, bridge and seal technique, fully covered self-expandable metallic stents (FCSEMSs), placement above the papilla

## Abstract

Background/Aims: Endoscopic management by endoscopic sphincterotomy with or without plastic stents or fully covered self-expandable metallic stents (FCSEMSs) is widely accepted as the current standard of care for postoperative bile leaks. Biliary stents are placed across the papilla, not above the papilla. We investigated the safety and effectiveness of the bridge-and-seal technique for bile leaks by the placement of FCSEMS above the papilla. Methods: This was a retrospective study of FCSEMS placement above the papilla for bile leaks between October 2016 and July 2021. FCSEMS was placed above the papilla to bridge and seal the leak. The main outcome measures were the resolution of bile leaks and adverse events. Results: Seven patients with postoperative bile leaks underwent FCSEMS above the papilla. The locations of bile leaks were 1 cystic duct remnant; 2 intrahepatic bile duct; 1 hepatic duct; 2 common bile duct and 1 anastomosis. The technical success rate of FCSEMS placement was 100%, and resolution of bile leaks was achieved in five patients (71.4%). All the adverse events were observed after FCSEMS removal; as follows: 1 moderate cholangitis; 2 mild post-ERCP pancreatitis; and 1 mild remnant cholecystitis. Conclusions: FCSEMS placement above the papilla can be a treatment option for postoperative bile leaks.

## 1. Introduction

Bile leaks are one of the major complications of hepatobiliary surgeries, such as cholecystectomy, liver transplantation, and hepatectomy. The reported incidence of postoperative bile leaks is 0.1–2% after cholecystectomy [1,2], 2–21% after liver transplantation [3,4], and 6–14% after hepatectomy [5]. With the widespread introduction of laparoscopy in the late 1980s, the incidence of bile leaks after cholecystectomy has increased: from 0.1–0.5% after open cholecystectomy to 0.8–2% after laparoscopic cholecystectomy.

Endoscopic retrograde cholangiopancreatography (ERCP) with endoscopic sphincterotomy [6], biliary stents [7] or its combination is the current standard treatment for postoperative bile leaks. Recently, the effectiveness of fully covered self-expandable metallic stents (FCSEMSs) was also reported [8,9,10]. A meta-analysis suggested that endoscopic sphincterotomy with leak-bridging stents had the highest success rate for bile leaks [11], but it is sometimes difficult to bridge the leak using conventional FCSEMS for anatomical reasons.

Several studies have demonstrated the advantage of FCSEMSs placed above the papilla for the treatment of benign biliary stricture, another major complication of hepatobiliary surgeries [12,13,14]. However, the effectiveness of FCSEMSs above the papilla for bile leaks has not been investigated to date. Herein, we report the bridge-and-seal technique using FCSEMS placement above the papilla as a new technique for the management of postoperative bile leaks.

## 2. Materials and Methods

### 2.1. Patients

This is a retrospective analysis of consecutive patients who underwent FCSEMS placement above the papilla for postoperative bile leaks between October 2016 and July 2021 at our institution. There were no definite criteria for the insertion of FCSEMS, and the types of stents were selected at the discretion of the endoscopists. We collected data from our prospectively maintained database. This study was approved by the local ethics committee.

### 2.2. Details of the Procedure

The FCSEMS used in this study was BONASTENT M-Intraductal (Standard SciTech., Seoul, Korea). This FCSEMS was originally designed for intraductal temporary placement for benign biliary strictures; the stent was fully covered with a silicone membrane, and a 10-cm-long lasso was attached to the distal end for stent removal. The central portion of the stent had a central waist to prevent stent migration.

**Figure 1:**
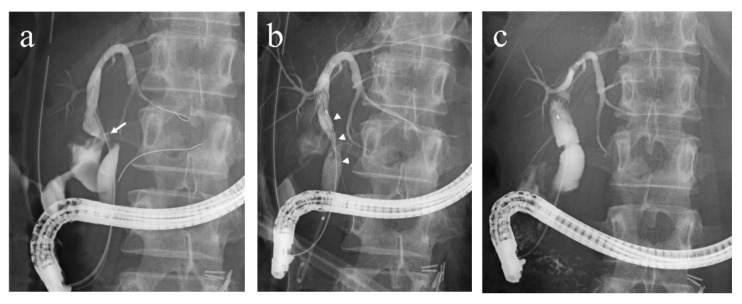
(**a**). After selective biliary cannulation, the bile leak was located by endoscopic retrograde cholangiography (ERC) (a white arrow). (**b**). A guidewire was placed over the leak, and the FCSEMS was deployed above the papilla under fluoroscopic guidance to seal the leak. The stent successfully bridged and sealed the leak (white arrowheads). (**c**). ERC following removal of the FCSEMS showed complete resolution of the bile leak.

Written informed consent was obtained prior to the procedure. A duodenoscope (TJF-260V, JF-260V; Olympus, Tokyo, Japan and ED-580T; Fujifilm, Tokyo, Japan) was intubated under moderate sedation. After selective biliary cannulation, the bile leak was located by endoscopic retrograde cholangiography (ERC) (Figure 1a). Bile leaks are classified as high grade when observed fluoroscopically prior to intrahepatic opacification [15]. A guidewire was placed across the site of the bile leak, and the FCSEMS was deployed above the papilla under fluoroscopic guidance to bridge and seal the leak (Figure 1b). Stent size was selected according to the bile duct diameter. A naso-biliary catheter was inserted concomitantly through the FCSEMS in cases with infection at bile leak (Figure 1c) and was removed a few days later. The FCSEMS was removed through the working channel of the duodenoscope by grasping the lasso with biopsy forceps at 3–6 months of stent placement. In principle, FCSEMS was removed after 3 months of stent placement to avoid difficulties in stent removal due to stent fracture or sludge formation inside the stent, as we previously reported in benign biliary strictures [13]. The resolution of the bile leaks was assessed by ERC. Adverse events were defined and graded according to the ASGE lexicon [16].

## 3. Results

### 3.1. Patient Characteristics

Seven patients (four males and three females) with a median age of 48 years old (range 25–89) were included in the study (Table 1). Bile leaks occurred following liver transplantation (LT) (*n* = 4 (3 living donor LT and 1 deceased donor LT)), open cholecystectomy for gallstone (*n* = 1), partial hepatectomy for metastatic liver tumor (*n* = 1), and radiofrequency ablation (RFA) for hepatocellular carcinoma (HCC) (*n* = 1).

Bile leaks were located at the cystic duct remnant (*n* = 1), the intrahepatic bile duct (*n* = 2), the hepatic duct (*n* = 1), the common bile duct (CBD) (*n* = 2) and the anastomosis (*n* = 1). In three cases, the bile leak was refractory to naso-biliary catheter placement and five cases were classified as high-grade leaks. The median interval between surgery and FCSEMS placement was 64 days (range, 3–212 days).

### 3.2. Effectiveness of FCSEMS above the Papilla for Bile Leaks

FCSEMS was successfully deployed to bridge the leak above the papilla in all seven patients (Table 2), with a technical success rate of 100%. No patient underwent endoscopic sphincterotomy (EST) prior to FCSEMS placement. In two cases with leaks at intrahepatic bile ducts, plastic stents were concomitantly placed in the contralateral bile duct to prevent cholangitis. A naso-biliary catheter was inserted through the FCSEMS in four patients and removed after 3 days (2–7 days) on average.

Removal of FCSEMS was attempted in six patients with a median indwelling time of 131.5 days (range, 74–189 days) and was successful in 100%. At FCSEMS removal, ERC showed complete resolution of bile leak in five patients. As a result, the bile leak resolution rate was achieved in 71.4%. There was no recurrence of bile leak after FCSEMS removal with a median follow-up period of 972.5 days (range, 55–1766 days) (Table 2).

In one patient after LDLT (Case #3), persistent bile leak was observed on cholangiogram after FCSEMS removal. Although biliary drainage was repeated, the patient developed liver failure due to uncontrolled cholangitis and liver abscesses and underwent liver transplantation again.

In one patient with bile leakage after RFA (Case #7), ERCP revealed a biloma with concomitant choledocho-duodenal fistula. Despite the disappearance of the biloma on CT scan, upper gastrointestinal endoscopy revealed persistent choledocho-duodenal fistula one month after FCSEMS placement. Given the rapid disease progression of advanced HCC, the decision was made to keep FCSEMS in place.

### 3.3. Adverse Events

A total of four ERCP-related adverse events were observed in three patients. Moderate cholangitis due to stent occlusion by bile sludge occurred in one patient (Case #3) three months after FCSEMS placement, which was managed by FCSEMS removal and temporary naso-biliary drainage placement. Two patients (Case #2, 3) suffered from mild pancreatitis after FCSEMS removal. One patient (Case #6) suffered from mild remnant cholecystitis after stent removal, which was managed conservatively with antibiotics. We did not encounter stent misplacement during ERCP or symptomatic stent migration thereafter, despite the lack of biliary strictures. However, one asymptomatic migration was observed at ERCP for stent removal (Case #2).

## 4. Discussion

Our case series suggested that FCSEMSs placement above the papilla was feasible for the treatment of postoperative bile leaks with a technical success rate of 100% and a bile leak resolution rate of 71.4%.

Conventionally, reducing the pressure inside the bile duct, either by sphincterotomy, plastic stent placement [17] or a combination of the two [7], is supposed to be an essential strategy for the treatment of bile leaks. Dechêne et al. reported that the success rate of plastic stent placement for leaks at the peripheral duct was as high as 88.9%, although that for leaks at the hilar or the common bile duct was 59.1% [18]. Baron et al. first reported the usefulness of FCSEMSs across the papilla for refractory bile leaks [10], and Canena et al. subsequently reported the superiority of FCSEMSs (100%) compared to the use of multiple plastic stents (65%) in refractory bile leaks [19]. However, pancreatitis due to the occlusion of the pancreatic duct orifice by FCSEMS is an inevitable problem [19].

Therefore, we attempted to “bridge and seal” the leak by deploying the FCSEMSs above the papilla. Recently, FCSEMSs placement above the papilla has been reported as a promising treatment option for patients with perihilar benign biliary strictures [13,14]. In our case series, even though the bile leak location of five cases (71.4%) was at perihilar areas or common bile ducts, we could identify the vanishment of bile leaks immediately after treatment and none of the seven patients suffered from pancreatitis after FCSEMS placement above the papilla, which could provide an advantage over the conventional FCSEMS placement across the papilla. Meanwhile, two patients suffered from pancreatitis after removal of the FCSEMS. In these cases, the traumatic removal of FCSEMSs is supposed to cause papillary edema, and EST may reduce the risk of pancreatitis after FCSEMS removal. In a prospective study of plastic stents above the papilla, EST did not affect the time to recurrent biliary obstruction [20]. Given the better outcomes of EST with a bridging plastic stent in a systematic review [11], it might be reasonable to add EST prior to FCSEMS placement above the papilla.

Regarding the indication of FCSEMS above the papilla for bile leaks, bile leaks at the hilar or the common bile duct and refractory cases to plastic stent treatment are suitable for FCSEMS. On the other hand, cases with the duct > 10 mm or <8 mm should not be an indication of FCSEMS.

In addition, there is no difference in the skills or time needed to insert FCSEMS between, above and across the papilla. The indication of FCSEMS above the papilla for bile leaks is that the bile leak location ranges from the upper to the middle bile duct. The advantages of this technique may be a reduced risk of pancreatitis and cholangitis caused by food impaction.

There are some limitations to this case series. The retrospective nature of our study, with a small sample size, is the major limitation. The resolution of bile leaks was not achieved in two cases with high-grade leaks, but further evaluation is warranted to evaluate the safety and efficacy of leak-sealing FCSEMS placement above the papilla for high-grade leaks. In addition, we inserted a temporary naso-biliary catheter in four patients, which might have affected the clinical outcomes in this study.

In conclusion, the bridge and seal technique using FCSEMSs above the papilla is a promising treatment option for postoperative bile leaks. A further prospective study with a larger cohort is needed to evaluate the efficacy of FCSEMS placement above the papilla compared with conventional plastic stents or FCSEMS placement across the papilla.

## Figures and Tables

**Table 1:** Patient characteristics.

Case	Age	Sex	Surgery	Time to Treatment after Surgery, Days	Location of the Bile Leak	Grade of Bile Leak	Prior Treatment
1	48	Male	LDLT	74	Anterior segmental branch	Low	Refractory to ENBD
2	25	Male	LDLT	212	Posterior segmental branch	High	Refractory to ENBD
3	34	Female	LDLT	42	Left hepatic duct	High	Refractory to ENBD
4	58	Male	DDLT	64	The anastomosis at common hepatic duct	High	Naive
5	51	Female	Partial hepatectomy	10	Upper Common bile duct	High	Naive
6	27	Female	Cholecystectomy	3	Cystic duct	Low	Naive
7	89	Male	RFA	95	Common bile duct	High	Naive

DDLT, deceased donor liver transplantation; ENBD, endoscopic naso-biliary drainage; LDLT, living donor liver transplantation; RFA, radiofrequency ablation.

**Table 2:** Details of the intervention and efficacy of the treatment.

Case	Stent Diameter, mm	Stent Length, mm	Concomitant ENBD Placement	Duration of Stent Placement, Days	Resolution of Bile Leak	Adverse Events	Follow-Up after FCSEMS Removal, Days
1	8	50	No	179	Yes	-	1028
2	8	40	Yes	105	Yes	Distal migration, pancreatitis	1766
3	10	50	No	74	No	Cholangitis, pancreatitis	1501
4	8	50	Yes	158	Yes	-	917
5	10	50	Yes	95	Yes	-	452
6	10	50	No	189	Yes	Remnant cholecystitis	55
7	8	50	Yes	166	No	-	N/A

FCSEMS, fully covered self-expandable metallic stent; ENBD, endoscopic naso-biliary drainage; N/A, not applicable.

## Data Availability

The datasets generated during and/or analyzed during the current study are not publicly available but are available from the corresponding author upon reasonable request.
